# Complete mitogenome of the streptophyte green alga *Coleochaete scutata* (Coleochaetophyceae)

**DOI:** 10.1080/23802359.2019.1693300

**Published:** 2019-11-22

**Authors:** Monique Turmel, Christian Otis, Claude Lemieux

**Affiliations:** Département de Biochimie, de Microbiologie et de Bio-Informatique, Institut de Biologie Intégrative et des Systèmes, Université Laval, Québec, Canada

**Keywords:** Coleochaetophyceae, horizontal transfers, mitochondrial genome, introns, phylogenomics, Streptophyta

## Abstract

We have sequenced the mitogenome of *Coleochaete scutata* strain SAG 110.80M. This mitogenome is the largest among the streptophyte green algae examined to date. At 242,024 bp, it is 4.3-fold larger than the mitogenome of *Chaetosphaeridium globosum*, the only other mitogenome available for the Coleochaetophyceae. This size difference is mainly explained by differences in the abundance of introns and in the length of intergenic regions containing vestiges of coding sequences thought to be of foreign origin. With 31 group I and 26 group II introns, the *C. scutata* mitogenome is the most intron-rich organelle genome known among streptophyte algae.

Previous studies on the mitogenomes of streptophyte green algae provided insights into the evolutionary dynamics of this genome within five of the six lineages recognized as classes (Turmel et al. 2007, 2013). Such information could not be obtained for the Coleochaetophyceae, which comprises one order and two families (Coleochaetaceae and Chaetosphaeridiaceae), because a single taxon (*Chaetosphaeridium globosum*) had been examined (Turmel et al. 2002). Here, we report the mitogenome sequence of *Coleochaete scutata* and compare it with its *Chaetosphaeridium* homolog.

The axenic strain SAG 110.80M of *C. scutata* was obtained from the Sammlung von Algenkulturen Göttingen and total DNA was isolated using the EZNA HP Plant Mini Kit of Omega Bio-Tek (Norcross, GA, USA). Paired-end reads were generated on the Illumina MiSeq sequencing platform by the ‘Plateforme d’Analyses Génomiques’ of Laval University. Reads (3.4 million) were assembled using Ray version 2.1.0 (Boisvert et al. 2010) and contigs were visualized, linked, and edited using CONSED version 22 (Gordon et al. 1998). Contigs of mitochondrial origin were identified by BLAST searches against a local database of organelle genomes. Gene annotations were performed as described by Turmel et al. (2017).

At 242,024 bp, the *C. scutata* mitogenome (GenBank: MN613583) is 4.3-fold larger than its *Chaetosphaeridium* homolog, but its gene repertoire is essentially the same. It possesses 67 distinct genes, three of which (*rrn5*, *trnMf*(cau) and *trnP*(ugg)) are duplicated or triplicated. The *rpl6* gene is missing from *C*. *scutata*, which represents the main difference in gene content between the two coleochaetophycean mitogenomes. Gene order is highly scrambled between these genomes. The larger size of the *C*. *scutata* mitogenome is mostly accounted for by a higher abundance of introns (57 *versus* 11 in *Chaetosphaeridium*) and by the presence of longer intergenic regions near *rns* (56.1 kb versus 8.3 kb). In both algae, the latter regions harbor ORFs showing similarities (*E-*value threshold of 1e-15) with recognized domains of phage/plasmid DNA primase and phage integrase. Considering that genes with these functions are not usually found in green algal mitochondria, we suggest that they were acquired by lateral transfers before the emergence of the Coleochaetophyceae. The *C*. *scutata* mitogenome contains 31 group I and 26 group II introns, a significant proportion of which (11 group I and 20 group II introns) encode LAGLIDADG homing endonucleases and reverse transcriptases/intron maturases, respectively. The *cox1* and *rnl* genes alone exhibit a total of 19 and 14 introns, respectively. Twenty-four of the *C*. *scutata* introns are inserted at sites where no introns have been observed in any of the currently available mitogenomes of streptophyte algae, supporting the view that mitochondrial introns proliferated in the Coleochaetaceae.

A maximum-likelihood phylogeny was inferred from 40 concatenated mitogenome-encoded proteins of 22 streptophyte taxa using RAxML version 8.2.3 (Stamatakis 2014) essentially as described in Turmel et al. (2013). The best-scoring tree is congruent with the streptophyte mitochondrial tree reported in the latter study (Figure 1). The two representatives of the Coleochaetophyceae are separated from one another by long branches but as expected, they fall in the same clade.

**Figure 1.**
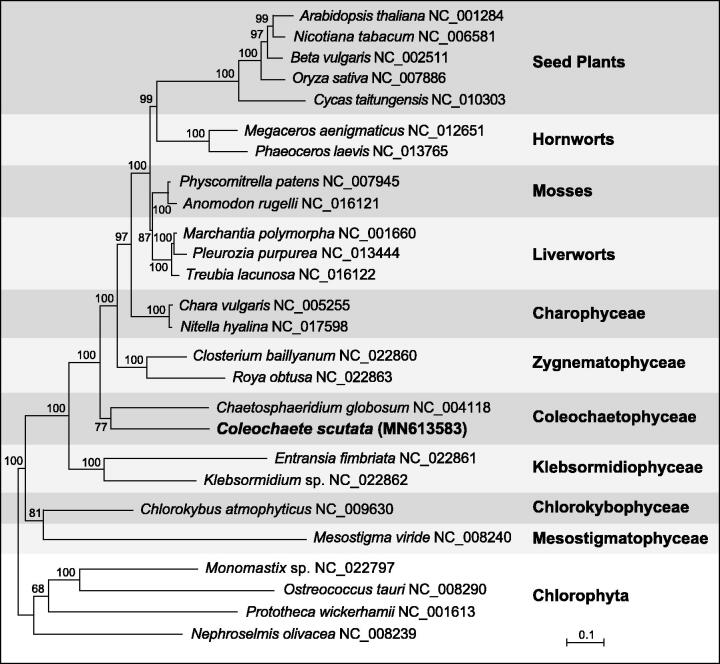
RAxML analysis of 40 concatenated mitogenome-encoded proteins from 22 streptophytes, including 12 land plants. The sequences of four chlorophytes were used to root the tree. The data set was partitioned by gene and each of the 40 partitions was applied to the GTR + Γ4 model. The figure shows the best-scoring tree, with bootstrap support values reported on the nodes. GenBank accession numbers for the mitogenomes of all taxa are provided. The scale bar denotes the estimated number of amino acid substitutions per site.
